# MSM 2010 Theme Monograph Psychopharmacology Today

**Published:** 2010

This theme monograph is called *Psychopharmacology Today*. It has some notable contributions on issues in psychopharmacology.

MSM 2010 is dedicated to the fond memory of Dr V.N. Bagadia, who headed our Hon International Editorial Advisory Board. See Dedication, “ Dr Bagadia, Sir, is no more”(p3).

Thomas L. Schwartz, M.D. Associate Professor of Psychiatry, SUNY Upstate Medical University, Syracuse, NY, USA writes an editorial on, “Psychopharmacology today: where are we and where do we go from here?”(p6).

Ajai R. Singh, MD. Editor, *Mens Sana Monographs*, writes the second editorial on, “Modern medicine: towards prevention, cure, well-being and longevity”(p17).

Sannidhya Varma, Himanshu Sareen and J.K. Trivedi from CSM Medical University, Lucknow, India, write on, “The Geriatric Population and Psychiatric Medication”(p30).

Amresh Shrivastava from The University of Western Ontario, Department of Psychiatry and Megan E. Johnston from University of Toronto, Department of Psychology, write on, “Weight Gain in Psychiatric Treatment: Risks, Implications, and Strategies for Prevention and Management”(p53).

Avinash De Sousa, M.D. Consultant Psychiatrist & Psychotherapist, Mumbai, writes on, “The Pharmacotherapy of Alcohol Dependence: A State of the Art Review”(p69).

Late B. M. Tripathi, M.D. Professor, Department of Psychiatry and National Drug Dependence and Treatment Center, All India Institute of Medical Sciences, and Pradipta Majumder, MBBS, Resident, Department of Psychiatry, All India Institute of Medical Sciences, New Delhi, write on, “Lactating Mother and Psychotropic Drugs”(p83).

K.S. Latha PhD. Associate Professor, Department of Psychiatry, KMC Hospital, Manipal University, writes on, “The Noncompliant Patient in Psychiatry: The Case For and Against Covert/Surreptitious Medication”(p96).

Neha Khetrapal, [M.A. Cognitive Science], Centre of Excellence “Cognitive Interaction Technology” (CITEC) And Faculty of Psychology & Sport Sciences, University of Bielefeld, Germany, writes for *The Looking Glass* on, “The Overlap of Autism and Seizures: Understanding Cognitive Comorbidity”(p122).

Leemon McHenry, Lecturer in Philosophy, California State University, Northridge, USA, writes in Journalology on, “Of Sophists and Spin-Doctors: Industry-Sponsored Ghostwriting and the Crisis of Academic Medicine”(p129).

Chittaranjan Andrade, M.D. Professor, Department of Psychopharmacology, National Institute of Mental Health and Neurosciences, Bangalore, India, writes a book review, “*MSM* Book Review 2010. *Stahl’s Essential Psychopharmacology: Neuroscientific Basis and Practical Applications*”(p146).

With this issue, we introduce flowchart for every paper as a figure. This gives a bird’s eye-view of the write-up. Another justification for you to read the article. Hopefully.

And also hope MSM 2010 provides stimulating reading to the discerning reader.

